# Supplementary material for: Social calls in humpback whale mother-calf groups off Sainte Marie breeding ground (Madagascar, Indian Ocean)
Source: PeerJ. 2022 Aug 16;10:e13785. doi: 10.7717/peerj.13785 (PMC9390327; doi:10.7717/peerj.13785)
Supplement: Supplemental Information 1 — (*) call types qualitatively similar to previously described call types. [file peerj-10-13785-s001.docx]

| LOW-FREQUENCY SOUNDS (LF) | | |
| --- | --- | --- |
| **100 Hz (*N* total = 303, *N* groups = 7)** |  |  |
|  | Mean±SD (Mi - Ma) | *N* |
| Dur | 0.52±0.26 (0.1 - 1.76) | 303 |
| Flow | 96±17 (50 - 190) | 244 |
| Fhigh | 159±23 (110 - 360) | 244 |
| Fexc | 63±15 (30 - 170) | 240 |
| Fmax | 131±25 (93 - 186) | 151 |
| Q25 | 129±39 (72 - 350) | 151 |
| Q50 | 198±228 (94 - 1798) | 151 |
| Q75 | 385±701 (99 - 3936) | 151 |
| Bdw | 17±11 (3 - 64) | 151 |
| F0 | 128±31 (55 - 186) | 68 |
| **Bass (*N* total = 84, *N* groups = 8)** |  |  |
|  | Mean±SD (Mi - Ma) | *N* |
| Dur | 1.22±0.79 (0.01 - 3.63) | 84 |
| Fmax | 46±5 (20 - 55) | 57 |
| Q25 | 39±10 (12 - 51) | 62 |
| Q50 | 59±26 (17 - 153) | 62 |
| Q75 | 281±412 (49 - 2733) | 62 |
| Bdw | 6±6 (1 - 46) | 57 |
| F0 | 36±12 (16 - 44) | 8 |
| **Boom (*N* total = 210, *N* groups = 4)** |  |  |
|  | Mean±SD (Mi - Ma) | *N* |
| Dur | 0.27±0.08 (0.1 - 0.56) | 210 |
| Flow | 54±24 (30 - 140) | 210 |
| Fhigh | 103±27 (70 - 180) | 210 |
| Fexc | 49±11 (20 - 90) | 202 |
| Fmax | 85±25 (52 - 150) | 156 |
| Q25 | 96±61 (55 - 461) | 156 |
| Q50 | 177±303 (61 - 2084) | 156 |
| Q75 | 353±771 (70 - 3897) | 156 |
| Bdw | 17±7 (3 - 46) | 156 |
| F0 | 74±28 (29 - 134) | 62 |
| **Gru (*N* total = 64, *N* groups = 6)** |  |  |
|  | Mean±SD (Mi - Ma) | *N* |
| Dur | 0.19±0.09 (0.05 - 0.48) | 64 |
| Flow | 125±64 (70 - 180) | 4 |
| Fhigh | 175±87 (100 - 250) | 4 |
| Fexc | 50±23 (30 - 70) | 4 |
| Fmax | 56±36 (10 - 164) | 42 |
| Q25 | 62±38 (17 - 216) | 42 |
| Q50 | 213±391 (29 - 1751) | 42 |
| Q75 | 881±864 (17 - 3058) | 42 |
| Bdw | 29±26 (5 - 170) | 42 |
| F0 | 41 | 1 |
| **Snort (*N* total = 56, *N* groups = 6)*** |  |  |
|  | Mean±SD (Mi - Ma) | *N* |
| Dur | 0.19±0.16 (0.05 - 0.9) | 56 |
| Fmax | 73±56 (17 - 280) | 43 |
| Q25 | 85±50 (41 - 230) | 43 |
| Q50 | 188±168 (46 - 846) | 43 |
| Q75 | 533±483 (102 - 2049) | 43 |
| Bdw | 44±61 (5 - 363) | 43 |
| F0 | 90±1 (90 - 91) | 2 |
| **Burp (*N* total = 18, *N* groups = 1)** |  |  |
|  | Mean±SD (Mi - Ma) | *N* |
| Dur | 0.47±0.21 (0.18 - 0.82) | 18 |
| Fmax | 29±11 (10 - 64) | 14 |
| Q25 | 33±9 (22 - 60) | 15 |
| Q50 | 77±16 (59 - 114) | 15 |
| Q75 | 227±116 (117 - 445) | 15 |
| Bdw | 10±5 (4 - 20) | 14 |
| F0 | 31±6 (27 - 35) | 2 |
| **Guttural sound (*N* total = 13, *N* groups = 1)** |  |  |
|  | Mean±SD (Mi - Ma) | *N* |
| Dur | 0.78±0.34 (0.55 - 1.89) | 13 |
| Fmax | 99±7 (92 - 107) | 6 |
| Q25 | 112±76 (56 - 245) | 9 |
| Q50 | 408±620 (13 - 1494) | 9 |
| Q75 | 861±1083 (11 - 2630) | 9 |
| Bdw | 16±5 (9 - 20) | 4 |
| **Thowp (*N* total = 35, *N* groups = 7)*** |  |  |
|  | Mean±SD (Mi - Ma) | *N* |
| Dur | 0.72±0.44 (0.11 - 1.63) | 35 |
| Fmax | 58±32 (19 - 105) | 9 |
| Q25 | 51±21 (23 - 96) | 12 |
| Q50 | 114±60 (57 - 246) | 12 |
| Q75 | 362±306 (128 - 1218) | 12 |
| Bdw | 20±14 (4 - 52) | 9 |
| F0 | 29 | 1 |
| **Wop (*N* total = 25, *N* groups = 8)*** |  |  |
|  | Mean±SD (Mi - Ma) | *N* |
| Dur | 0.3±0.18 (0.1 - 0.85) | 25 |
| Flow | 60 | 1 |
| Fhigh | 190 | 1 |
| Fexc | 130 | 1 |
| Fmax | 76±107 (23 - 413) | 12 |
| Q25 | 65±56 (38 - 251) | 13 |
| Q50 | 130±97 (50 - 439) | 13 |
| Q75 | 434±466 (58 - 1751) | 13 |
| Bdw | 27±16 (8 - 55) | 12 |
| F0 | 41 | 1 |
| **Bark (*N* total = 2, *N* groups = 1)*** |  |  |
|  | Mean±SD (Mi - Ma) | *N* |
| Dur | 0.33±0.03 (0.31 - 0.35) | 2 |
| Flow | 70 | 1 |
| Fhigh | 670 | 1 |
| Fexc | 600 | 1 |
| Fmax | 148±1 (147 - 149) | 2 |
| Q25 | 144±13 (134 - 153) | 2 |
| Q50 | 252±62 (208 - 295) | 2 |
| Q75 | 720±392 (442 - 997) | 2 |
| Bdw | 24±1 (23 - 24) | 2 |
| **Drum (*N* total = 115, *N* groups = 2)** |  |  |
|  | Mean±SD (Mi - Ma) | *N* |
| Dur | 0.11±0.05 (0.04 - 0.26) | 115 |
| Fmax | 157±70 (23 - 310) | 74 |
| Q25 | 145±55 (46 - 263) | 74 |
| Q50 | 285±153 (93 - 814) | 74 |
| Q75 | 877±522 (160 - 2824) | 74 |
| Bdw | 76±62 (14 - 290) | 74 |
| MID-FREQUENCY HARMONIC SOUNDS (MF) | | |
| **Groan (*N* total = 2, *N* groups = 1)*** |  |  |
|  | Mean±SD (Mi - Ma) | *N* |
| Dur | 5.01±2.05 (3.56 - 6.46) | 2 |
| Flow | 135±21 (120 - 150) | 2 |
| Fhigh | 460±71 (410 - 510) | 2 |
| Fexc | 325±49 (290 - 360) | 2 |
| **Downsweep (*N* total = 140, *N* groups = 5)** | |  |
|  | Mean±SD (Mi - Ma) | *N* |
| Dur | 0.67±0.32 (0.18 - 1.7) | 140 |
| Flow | 307±189 (110 - 2060) | 128 |
| Fhigh | 736±218 (250 - 2780) | 128 |
| Fexc | 429±130 (80 - 790) | 128 |
| Fmax | 392±304 (12 - 2090) | 44 |
| Q25 | 429±278 (217 - 2045) | 43 |
| Q50 | 654±315 (339 - 2255) | 43 |
| Q75 | 1608±602 (564 - 3026) | 43 |
| Bdw | 44±59 (6 - 287) | 42 |
| F0 | 135±64 (104 - 292) | 8 |
| **Woohoo (*N* total = 141, *N* groups = 5)** |  |  |
|  | Mean±SD (Mi - Ma) | *N* |
| Dur | 0.93±0.5 (0.28 - 2.56) | 141 |
| Flow | 230±52 (90 - 370) | 108 |
| Fhigh | 313±60 (160 - 440) | 108 |
| Fexc | 83±39 (10 - 280) | 108 |
| Fmax | 288±64 (204 - 431) | 28 |
| Q25 | 331±63 (215 - 435) | 28 |
| Q50 | 728±370 (319 - 1496) | 28 |
| Q75 | 2158±457 (1037 - 2921) | 28 |
| Bdw | 23±63 (3 - 344) | 28 |
| F0 | 246±82 (201 - 393) | 5 |
| **Trumpet (*N* total = 28, *N* groups = 3)*** |  |  |
|  | Mean±SD (Mi - Ma) | *N* |
| Dur | 1.63±0.39 (0.71 - 2.26) | 28 |
| Flow | 214±88 (100 - 430) | 24 |
| Fhigh | 294±109 (210 - 580) | 24 |
| Fexc | 80±34 (40 - 150) | 24 |
| Fmax | 372±131 (204 - 505) | 13 |
| Q25 | 509±173 (219 - 925) | 16 |
| Q50 | 1024±510 (485 - 1801) | 16 |
| Q75 | 2012±706 (890 - 2828) | 16 |
| Bdw | 17±10 (6 - 32) | 9 |
| F0 | 462 | 1 |
| **Heek (*N* total = 251, *N* groups = 9)** |  |  |
|  | Mean±SD (Mi - Ma) | *N* |
| Dur | 0.4±0.23 (0.08 - 1.3) | 251 |
| Flow | 223±277 (20 - 2340) | 94 |
| Fhigh | 369±322 (90 - 2710) | 94 |
| Fexc | 147±86 (30 - 380) | 93 |
| Fmax | 422±481 (41 - 2797) | 199 |
| Q25 | 505±442 (48 - 2588) | 199 |
| Q50 | 1087±685 (112 - 3232) | 199 |
| Q75 | 1955±911 (196 - 3928) | 199 |
| Bdw | 36±27 (5 - 175) | 197 |
| F0 | 160±8 (155 - 166) | 2 |
| **Whoop (*N* total = 112, *N* groups = 7)** |  |  |
|  | Mean±SD (Mi - Ma) | *N* |
| Dur | 0.78±0.34 (0.19 - 1.43) | 112 |
| Flow | 431±137 (110 - 720) | 94 |
| Fhigh | 656±176 (350 - 970) | 94 |
| Fexc | 206±95 (60 - 470) | 58 |
| Fmax | 499±138 (141 - 708) | 77 |
| Q25 | 515±179 (151 - 1557) | 77 |
| Q50 | 682±216 (8 - 1261) | 77 |
| Q75 | 1701±596 (536 - 3382) | 76 |
| Bdw | 38±64 (2 - 292) | 76 |
| F0 | 339±187 (126 - 703) | 16 |
| **Wiper (*N* total = 40, *N* groups = 1)** |  |  |
|  | Mean±SD (Mi - Ma) | *N* |
| Dur | 0.23±0.05 (0.11 - 0.34) | 40 |
| Flow | 282±129 (100 - 480) | 9 |
| Fhigh | 506±201 (220 - 830) | 9 |
| Fexc | 223±82 (110 - 350) | 9 |
| Fmax | 390±270 (65 - 1136) | 34 |
| Q25 | 349±173 (134 - 899) | 34 |
| Q50 | 702±301 (325 - 1801) | 34 |
| Q75 | 1371±438 (723 - 2824) | 34 |
| Bdw | 54±36 (14 - 164) | 34 |
| **Creek (*N* total = 4, *N* groups = 1)*** |  |  |
|  | Mean±SD (Mi - Ma) | *N* |
| Dur | 0.24±0.11 (0.14 - 0.34) | 4 |
| Flow | 410 | 1 |
| Fhigh | 730 | 1 |
| Fexc | 320 | 1 |
| Fmax | 374±141 (199 - 506) | 4 |
| Q25 | 805±798 (339 - 1995) | 4 |
| Q50 | 1434±1305 (544 - 3351) | 4 |
| Q75 | 2473±1191 (1535 - 4204) | 4 |
| Bdw | 111±88 (35 - 237) | 4 |
| HIGH-FREQUENCY HARMONIC SOUNDS (HF) | | |
| **Squeak (*N* total = 160, *N* groups = 7)*** |  |  |
|  | Mean±SD (Mi - Ma) | *N* |
| Dur | 0.29±0.14 (0.1 - 1.13) | 160 |
| Flow | 1052±160 (390 - 1600) | 156 |
| Fhigh | 1283±118 (990 - 1830) | 156 |
| Fexc | 231±137 (70 - 770) | 156 |
| Fmax | 1202±353 (58 - 3676) | 76 |
| Q25 | 1158±203 (875 - 2712) | 77 |
| Q50 | 1432±367 (984 - 3545) | 77 |
| Q75 | 2626±480 (1751 - 3760) | 77 |
| Bdw | 27±19 (7 - 124) | 76 |
| F0 | 606±419 (62 - 1384) | 23 |
| **Ascending shriek (*N* total = 42, *N* groups = 4)*** | |  |
|  | Mean±SD (Mi - Ma) | *N* |
| Dur | 1.27±0.81 (0.21 - 4.11) | 42 |
| Flow | 968±670 (260 - 2020) | 40 |
| Fhigh | 1702±499 (1190 - 3010) | 40 |
| Fexc | 733±470 (70 - 1550) | 40 |
| Fmax | 1753±418 (1179 - 2418) | 18 |
| Q25 | 1645±395 (889 - 2068) | 18 |
| Q50 | 2089±357 (1310 - 2512) | 18 |
| Q75 | 3025±688 (2286 - 4208) | 18 |
| Bdw | 22±24 (3 - 104) | 18 |
| AMPLITUDE MODULATED SOUNDS (AM) | | |
| **Door (*N* total = 5, *N* groups = 1)** |  |  |
|  | Mean±SD (Mi - Ma) | *N* |
| Dur | 3.39±0.35 (2.83 - 3.78) | 5 |
| Fmax | 844 | 1 |
| Q25 | 824 | 1 |
| Q50 | 1515 | 1 |
| Q75 | 2849 | 1 |
| **Whine (*N* total = 5, *N* groups = 1)** |  |  |
|  | Mean±SD (Mi - Ma) | *N* |
| Dur | 2.42±0.87 (1.05 - 3.06) | 5 |
| **Trill (*N* total = 104, *N* groups = 5)*** |  |  |
|  | Mean±SD (Mi - Ma) | *N* |
| Dur | 2.29±0.91 (0.98 - 5.02) | 104 |
| Flow | 275±85 (100 - 430) | 18 |
| Fhigh | 409±86 (300 - 580) | 18 |
| Fexc | 134±77 (40 - 270) | 18 |
| Fmax | 333±74 (233 - 487) | 23 |
| Q25 | 353±96 (243 - 641) | 25 |
| Q50 | 609±177 (345 - 937) | 25 |
| Q75 | 1771±634 (921 - 3828) | 25 |
| **Bug sound (*N* total = 20, *N* groups = 1)** |  |  |
|  | Mean±SD (Mi - Ma) | *N* |
| Dur | 3.08±0.63 (1.09 - 4.2) | 20 |
| Flow | 244±68 (110 - 310) | 10 |
| Fhigh | 363±72 (260 - 490) | 10 |
| Fexc | 119±43 (70 - 190) | 10 |
| Fmax | 342±44 (294 - 401) | 4 |
| Q25 | 371±76 (281 - 499) | 7 |
| Q50 | 653±250 (369 - 1020) | 7 |
| Q75 | 1290±325 (880 - 1701) | 7 |
| F0 | 295 | 1 |
| **AM grunt (*N* total = 33, *N* groups = 6)*** |  |  |
|  | Mean±SD (Mi - Ma) | *N* |
| Dur | 0.38±0.32 (0.06 - 1.78) | 33 |
| Fmax | 168±320 (11 - 1511) | 20 |
| Q25 | 164±186 (23 - 884) | 20 |
| Q50 | 535±559 (49 - 1792) | 20 |
| Q75 | 1203±1055 (111 - 3700) | 20 |
| Bdw | 30±42 (7 - 199) | 20 |
| PULSED SOUNDS (PS) | | |
| **Fry (*N* total = 11, *N* groups = 2)** |  |  |
|  | Mean±SD (Mi - Ma) | *N* |
| Dur | 0.78±0.3 (0.45 - 1.43) | 11 |
| Fmax | 31±14 (20 - 66) | 11 |
| Q25 | 69±41 (29 - 155) | 11 |
| Q50 | 320±206 (58 - 600) | 11 |
| Q75 | 1051±659 (259 - 2524) | 11 |
| Bdw | 10±5 (4 - 21) | 11 |
| PR | 23±3 (20 - 27) | 11 |
| **Bubble sound (*N* total = 2, *N* groups = 1)** | |  |
|  | Mean±SD (Mi - Ma) | *N* |
| Dur | 0.75±0.23 (0.59 - 0.91) | 2 |
| Fmax | 39±1 (38 - 40) | 2 |
| Q25 | 62±12 (54 - 71) | 2 |
| Q50 | 200±80 (143 - 256) | 2 |
| Q75 | 499±281 (300 - 698) | 2 |
| Bdw | 10±5 (7 - 14) | 2 |
| PR | 19 | 1 |
| **Moped (*N* total = 4, *N* groups = 1)** |  |  |
|  | Mean±SD (Mi - Ma) | *N* |
| Dur | 3.8±1.42 (2.91 - 5.9) | 4 |
| Fmax | 403±37 (353 - 439) | 4 |
| Q25 | 306±102 (210 - 403) | 4 |
| Q50 | 639±178 (423 - 802) | 4 |
| Q75 | 1457±231 (1120 - 1640) | 4 |
| Bdw | 2±1 (1 - 2) | 4 |
| F0 | 192±20 (170 - 217) | 4 |
| PR | 6±0 (6 - 7) | 4 |
| **Gloop (*N* total = 4, *N* groups = 1)** |  |  |
|  | Mean±SD (Mi - Ma) | *N* |
| Dur | 3.02±1.46 (1.08 - 4.58) | 4 |
| Fmax | 472±238 (314 - 827) | 4 |
| Q25 | 316±32 (286 - 358) | 4 |
| Q50 | 487±123 (407 - 668) | 4 |
| Q75 | 821±62 (753 - 903) | 4 |
| Bdw | 3±2 (1 - 6) | 4 |
| PR | 11±2 (9 - 13) | 4 |
